# Mitochondrial iron-sulfur cluster dysfunction in neurodegenerative disease

**DOI:** 10.3389/fphar.2014.00029

**Published:** 2014-03-03

**Authors:** Grazia Isaya

**Affiliations:** Department of Pediatric & Adolescent Medicine and Mayo Clinic Children’s CenterMayo Clinic, Rochester, MN, USA

**Keywords:** Friedreich ataxia, Parkinson disease, aging, mitochondria, iron–sulfur clusters, oxidative damage, anti-oxidants, iron-chelators

## Abstract

Growing evidence supports a role for mitochondrial iron metabolism in the pathophysiology of neurodegenerative disorders such as Friedreich ataxia (FRDA) and Parkinson disease (PD) as well as in the motor and cognitive decline associated with the aging process. Iron–sulfur enzyme deficits and regional iron accumulation have been observed in each of these conditions. In spite of significant etiological, clinical and pathological differences that exist between FRDA and PD, it is possible that defects in mitochondrial iron–sulfur clusters (ISCs) biogenesis represent a common underlying mechanism leading to abnormal intracellular iron distribution with mitochondrial iron accumulation, oxidative phosphorylation deficits and oxidative stress in susceptible cells and specific regions of the nervous system. Moreover, a similar mechanism may contribute to the age-dependent iron accumulation that occurs in certain brain regions such as the globus pallidus and the substantia nigra. Targeting chelatable iron and reactive oxygen species appear as possible therapeutic options for FRDA and PD, and possibly other age-related neurodegenerative conditions. However, new technology to interrogate ISC synthesis in humans is needed to (i) assess how defects in this pathway contribute to the natural history of neurodegenerative disorders and (ii) develop treatments to correct those defects early in the disease process, before they cause irreversible neuronal cell damage.

## THE NATURAL HISTORY OF NEURODEGENERATIVE DISEASE

**FIGURE 1:**
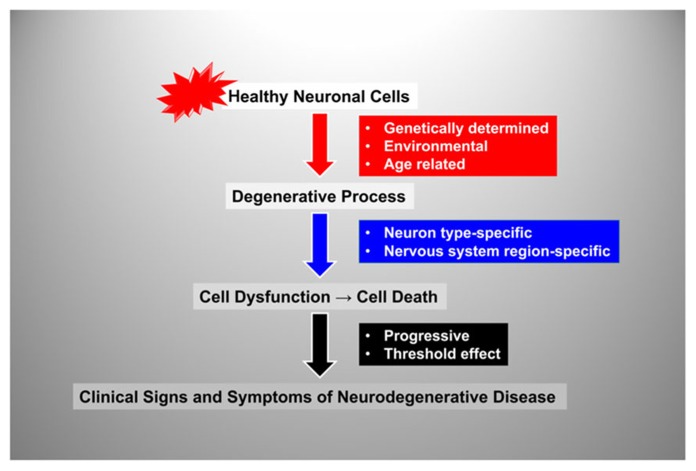
**Flow-chart representation of the steps and factors involved in the natural history of neurodegenerative disorders.** See text for details.

Although neurodegenerative disorders can present at different ages and with a broad variety of symptoms, their natural histories can be recapitulated by a few common steps (**Figure 1**): Normally developed and overall healthy neuronal cells are exposed to an insult that initiates the neurodegenerative process; this leads to progressive neuronal cell dysfunction and death, which ultimately leads to clinical signs and symptoms of neurological impairment. However, each of these common steps is influenced by different disease-specific factors. The initiating insult can be genetically-determined or environmentally-determined or result from a combination of both genetic and environmental factors. In addition, for many neurodegenerative disorders aging is a consistently important risk factor (Zecca et al., 2004b). The neurodegenerative process is often neuron-type specific–for example, motor neurons are exquisitely affected in amyotrophic lateral sclerosis, sensory neurons in Friedreich ataxia (FRDA), and dopaminergic neurons in Parkinson disease (PD) (Koeppen, 2011; Oshiro et al., 2011). In all cases, the neurodegenerative process is relentlessly progressive and there is a threshold for neurological manifestations, meaning that a critical mass of affected neuronal cells will have to become dysfunctional and/or die in order for clinical signs and symptoms to become apparent. The rate of progression and the threshold may vary depending on the cell type and respective brain region affected as well as additional factors that may influence disease severity (e.g., the presence of concomitant cardiac insufficiency and skeletal muscle weakness in FRDA). Importantly, before the threshold is reached there is a pre-symptomatic period during which the degenerative process is already advancing.

**FIGURE 2:**
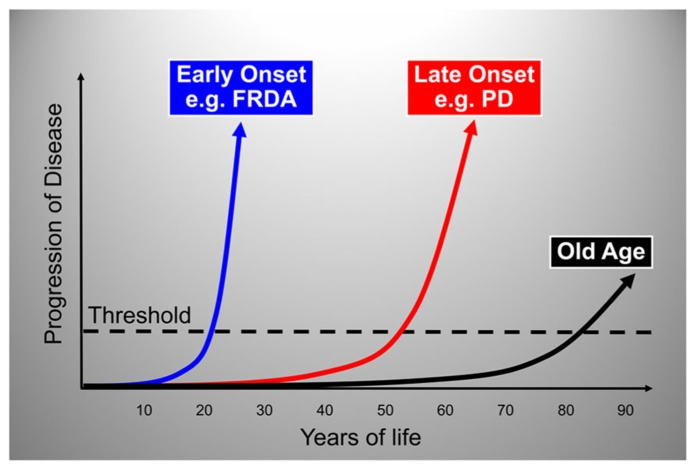
**Graphic representation of the three main modalities in the natural history of neurodegenerative disease.** The rate of neurodegenerative disease progression is plotted as a function of age with an arbitrary threshold for appearance of clinical signs and symptoms. The plots show typical rates of progression for old age-related, late-onset and early-onset neurodegenerative disorders. The portion of each plot below the threshold represents the pre-symptomatic period during which the degenerative process is already active. FRDA, Friedreich ataxia; PD, Parkinson disease.

By plotting disease progression as a function of age with an arbitrary threshold for appearance of clinical signs and symptoms we can define three main modalities in the natural history of neurodegenerative disease (**Figure 2**). In most individuals there are slowly progressing, age-related degenerative changes that become clinically apparent only at an advanced age, manifesting as a decline in motor and cognitive performance after the 7th or 8th decade of life. In addition, there are conditions in which neurodegeneration occurs at a much faster pace with onset around the 6th,5th, or 4th decade or life, and others in which onset is much earlier, in the 2nd or even the 1st decade of life (**Figure 2**).

Below we will address the question as to whether and how defects in mitochondrial iron metabolism may contribute to these clinical scenarios. Indeed, there are at least two conditions, FRDA and PD, that provide paradigms for the link between mitochondrial iron dysfunction and, respectively, early-onset and late-onset neurodegeneration (Zecca et al., 2004a; Koeppen et al., 2007; Koeppen, 2011) [reviewed in (Horowitz and Greenamyre, 2010; Oshiro et al., 2011; Vaubel and Isaya, 2013)]. **Table 1** summarizes published evidence which together suggests that FRDA and PD share certain common aspects, also observed in the aging brain. These aspects include (i) regional iron accumulation in specific regions of the central and/or peripheral nervous systems (Zecca et al., 1996; Bartzokis et al., 2004; Boddaert et al., 2007; Oakley et al., 2007; Koeppen, 2011); (ii) cellular iron re-distribution within affected cell types that may result in mitochondrial iron accumulation and iron-catalyzed Fenton chemistry (Zecca et al., 2004a; Whitnall et al., 2008; Mastroberardino et al., 2009); and (iii) the presence of iron–sulfur enzyme deficits (Rotig et al., 1997; Longo et al., 1999; Betarbet et al., 2000) [reviewed in (Xu et al., 2010; Gille and Reichmann, 2011; Vaubel and Isaya, 2013; **Table 1**)]. This evidence supports the view that a common underlying mechanism involving iron metabolism–namely, defects in the biogenesis of mitochondrial iron–sulfur clusters (ISCs) and related enzymes–contributes to FRDA, PD and the aging process, in spite of these three conditions being clinically, pathologically and etiologically very different from one another. Several excellent recent review articles recapitulate the current understanding of the molecules and mechanisms involved in ISC synthesis as well as the roles of iron dysregulation in the pathophysiology of FRDA, PD, and the aging process. Here we will review and discuss primarily the roles of ISC dysfunction in these conditions.

**Table 1:** Indicators of mitochondrial Fe–S cluster dysfunction in FRDA, PD, and aging.

	FRDA^*^	PD^**^	Aging^***^
Regional iron accumulation	Dorsal root ganglia, Nucleus dentatus, Heart	Substantia nigra	Globus pallidus, Substantia nigra, Skeletal muscle
Cellular iron redistribution, mitochondrial iron accumulation, HO•	+	+	+
Fe–S enzyme deficits	Generalized	Complex I	Mitochondrial aconitase

* (Rotig et al., 1997; Koeppen, 2011);

** (Horowitz and Greenamyre, 2010);

*** (Bota et al., 2002; Xu et al., 2010).

## IRON–SULFUR CLUSTER SYNTHESIS AND NEURODEGENERATIVE DISEASE

**FIGURE 3:**
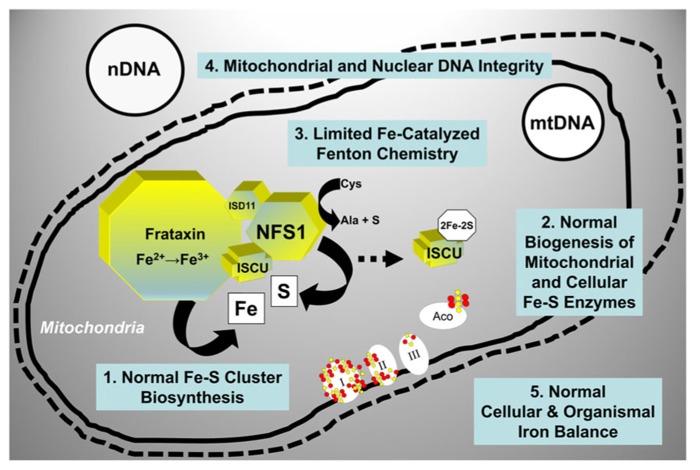
**Molecular components involved in the initial step of mitochondrial ISC synthesis and their biological roles.** When mitochondrial ISC synthesis functions normally, vital enzyme activities are maintained throughout the cell, iron-catalyzed oxidative damage is limited, and there is a balance between iron uptake and iron utilization. See text for additional details. NFS1, cysteine desulfurase; ISD11, adaptor protein required for NFS1 stability; ISCU, scaffold protein; I, II, III, respiratory chain complexes I, II, and III; Aco, mitochondrial aconitase; mtDNA, mitochondrial DNA; nDNA, nuclear DNA.

Mitochondria across eukaryotes contain a machinery that is responsible for the biogenesis of ISC inside mitochondria (Schilke et al., 1999; Muhlenhoff et al., 2002), and that also somehow regulates the assembly of ISC in other cellular compartments (Kispal et al., 1999; Gerber et al., 2004; Pondarre et al., 2006; Martelli et al., 2007). ISC-dependent enzymes are present in the mitochondria, the cytoplasm and the nucleus where they participate in such processes as the citric acid cycle and the electron transport chain, ribosome biogenesis, and nuclear DNA synthesis and repair among others [reviewed in (Ye and Rouault, 2010; Lill et al., 2012)]. The core machinery that catalyzes the initial step in ISC assembly in the mitochondrial matrix consists of cysteine desulfurase (NFS1), a cysteine desulfurase that generates elemental sulfur; frataxin, an iron-binding protein that provides elemental iron and also stimulates NFS1 activity; and scaffold protein (ISCU), a scaffold protein upon which [2Fe–2S] and [4Fe–4S] clusters are initially assembled before being transferred to the appropriate enzymes [reviewed in (Ye and Rouault, 2010; Lill et al., 2012; Vaubel and Isaya, 2013)]. When this process functions normally, vital enzyme activities are maintained throughout the cell, iron-catalyzed oxidative damage is limited, and there is a balance between cellular iron uptake and mitochondrial iron utilization (**Figure 3**).

The consequences of defects in ISC synthesis have been extensively studied in a number of *S. cerevisiae* mutants in which this process was genetically impaired [although only partially since a complete loss of ISC synthesis is not compatible with life across eukaryotes (Cossee et al., 2000; Lill and Kispal, 2000; Kispal et al., 2005)]. These mutants have consistently shown a series of key mitochondrial and cellular features. Within mitochondria, reduced formation of ISC leads to an increase in the fraction of labile iron that leads to higher rates of Fenton chemistry resulting in loss of mitochondrial DNA integrity and overall loss of oxidative phosphorylation (Knight et al., 1998; Li et al., 1999; Karthikeyan et al., 2003). Simultaneously, the cell responds to reduced mitochondrial ISC synthesis with a rapid increase in cellular iron uptake and intracellular re-distribution of iron that is depleted in the cytoplasm but continues to accumulate in mitochondria until it precipitates out of solution as an amorphous mineral (Babcock et al., 1997; Knight et al., 1998; Li et al., 1999; Chen et al., 2004). These features hold true in multicellular organisms including humans as we will see in the specific cases of FRDA and PD.

## FRIEDREICH ATAXIA

FRDA is an autosomal recessive disease and the most common genetically-determined ataxia that affects approximately 1:40,000 individuals in the Caucasian population [for recent reviews see (Pandolfo, 2009; Koeppen, 2011)]. Patients are healthy at birth and remain largely asymptomatic for the first 5–10 years of life but then begin to present progressive neurological impairment and additional problems including cardiac disease, muscle weakness, skeletal deformities, vision and hearing loss, and diabetes. Patients eventually become wheelchair-bound and most often die of cardiac failure in the 2nd or 3rd decade of life. Certain regions of the central (cerebellum and spinal cord) and peripheral (dorsal root ganglia and their nerves) nervous systems as well as the heart, skeletal muscles, skeleton, and endocrine pancreas are affected (Koeppen, 2011). This early-onset, very dramatic clinical and pathological progression results in most patients from reduced levels of a mitochondrial iron-binding protein called frataxin (Campuzano et al., 1996). The biochemical properties of frataxin include the ability to bind iron, the ability to donate iron to other iron-binding proteins, and the ability to oligomerize, store iron and control iron redox chemistry [reviewed in (Bencze et al., 2006)]. Through these properties, frataxin plays key roles in different iron-dependent pathways (primarily, although not exclusively, ISC synthesis) and is therefore critical for mitochondrial iron metabolism and overall cellular iron homeostasis and antioxidant protection [reviewed in (Wilson, 2006; Vaubel and Isaya, 2013)].

### MITOCHONDRIAL AND OTHER CELLULAR CONSEQUECES OF FRATAXIN DEFICIENCY

Reduced levels of frataxin in FRDA mouse models and human patients result in defects in ISC enzymes even before mitochondrial iron accumulation becomes detectable (Puccio et al., 2001; Stehling et al., 2004). However, iron dysregulation is an early effect of frataxin depletion, which increases the fraction of labile redox-active iron inside mitochondria (Wong et al., 1999) leading to progressive accumulation of oxidative damage (Whitnall et al., 2012). In addition, in mouse heart the lack of frataxin results in gene expression changes leading to down-regulation of proteins involved in mitochondrial ISC synthesis, heme synthesis, and iron storage as well as proteins involved in cellular and mitochondrial iron uptake, which collectively lead to intracellular iron redistrbution and progressive mitochondrial iron accumulation (Huang et al., 2009). All of these effects ultimately result in progressive impairment of energy metabolism and accumulation of oxidative damage [reviewed in (Pandolfo, 2006; Wilson, 2006)]. There are also additional changes outside of mitochondria affecting pathways involved in antioxidant, metabolic, and inflammatory responses, which are believed to contribute to disease progression [(Pianese et al., 2002; Coppola et al., 2009; Lu et al., 2009; Paupe et al., 2009; Sparaco et al., 2009; Wagner et al., 2012); reviewed in (Pandolfo, 2012)].

## PARKINSON DISEASE

PD is a common neurodegenerative disease affecting ~1:800 individuals between the 5th and 7th decade of life with a median survival from diagnosis of about 15 years. The degenerative process exquisitely affects dopaminergic neurons in the substantia nigra and clinically leads to progressive motor deficits including bradykinesia, rigidity, resting tremor, and postural instability as well as a variety of non-motor symptoms in the late stages of the disease. Age is the main risk factor for PD although susceptibility genes and environmental toxins are also implicated in the pathophysiology [reviewed in (Lees et al., 2009)].

### IRON DYSREGULATION CONTRIBUTES TO PD

**FIGURE 4:**
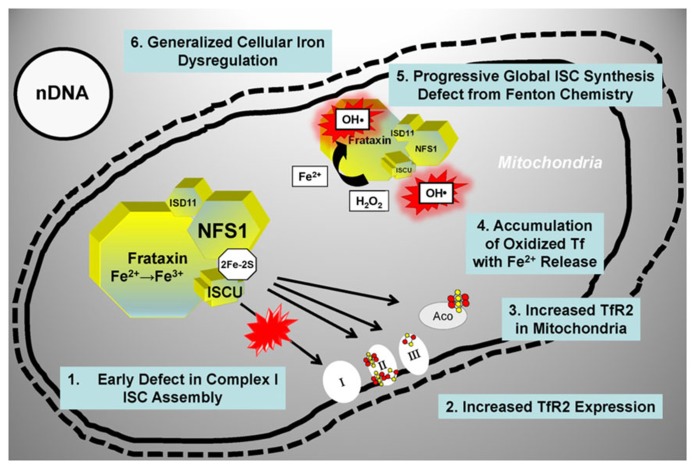
** Proposed model for a role of ISC synthesis defects in PD progression.** See text for details. Tf, transferrin; TfR2, transferrin receptor; other abbreviations are as in the legend for **Figure 3**.

A significant body of data [reviewed in (Mastroberardino et al., 2009)] supports a role for a defect in ISC synthesis in PD: (i) mitochondrial impairment and oxidative stress are involved in the pathophysiology of PD (Greenamyre and Hastings, 2004); (ii) partial inhibition of Complex I of the respiratory chain (which includes several ISC-containing subunits) recapitulates many features of PD (Betarbet et al., 2000; Przedborski et al., 2001); (iii) iron levels are increased in the substantia nigra and within substantia nigra dopaminergic neurons of PD patients (Berg and Hochstrasser, 2006; Oakley et al., 2007); (iv) iron chelation protects substantia nigra neurons in animal models of PD (Ben-Shachar et al., 1991; Kaur et al., 2003) as well as PD patients (Devos et al., 2013). Mastroberardino et al. have described a novel pathway of iron transport to mitochondria of substantia nigra dopaminergic neurons involving transferrin and the transferrin receptor 2 (Mastroberardino et al., 2009). This pathway normally delivers transferrin-bound iron to mitochondria and to Complex I of the respiratory chain. In PD, however, there is an induction of transferrin receptor 2 expression and accumulation of oxidized transferrin inside mitochondria, which results in the release of labile ferrous iron from transferrin and the generation of hydroxyl radicals via Fenton chemistry (Mastroberardino et al., 2009; Horowitz and Greenamyre, 2010). A concomitant reduction in mitochondrial ISC synthesis has been postulated (Horowitz and Greenamyre, 2010), and could probably account for the cascade of events described above in at least two ways (**Figure 4**). Early on, a specific defect in the synthesis and/or delivery of ISC cofactors needed for Complex I activity could induce the transferring receptor 2 pathway, thereby triggering the increase in cellular iron uptake and the ensuing mitochondrial iron accumulation with iron-catalyzed oxidative damage. Under these conditions, the need to handle redox-active iron could likely make the ISC assembly machinery especially susceptible to oxidative damage from radicals generated via Fenton chemistry. Thus, progressive accumulation of oxidative damage could later lead to a more generalized ISC synthesis defect further enhancing cellular iron dysregulation in dopaminergic neurons (**Figure 4**).

## IRON ACCUMULATION IN THE AGING BRAIN

**FIGURE 5:**
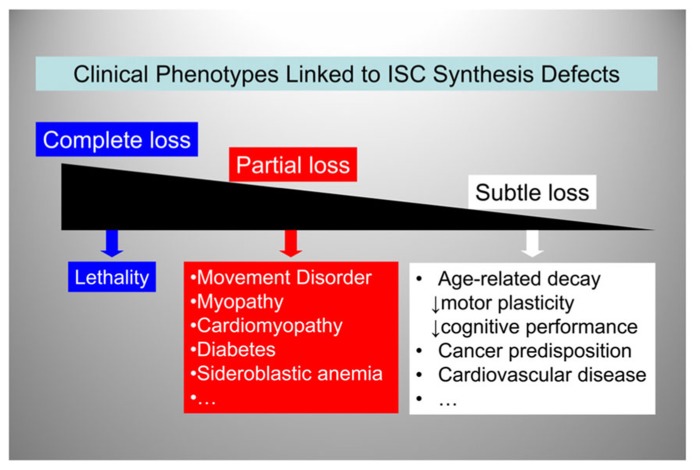
**A spectrum of clinical phenotypes may be linked to ISC synthesis defects.** ISC synthesis defects of decreasing severity lead to a variety of clinical phenotypes ranging from embryonic lethality to increased predisposition to age-related disorders.

So far we have reviewed FRDA, where a defect in ISC synthesis is clearly associated with disease pathogenesis early on in the disease process, and PD, where a defect in ISC synthesis may be involved at both an early and a late stage of disease progression. Could a reduction in ISC synthesis also contribute to age-dependent iron accumulation in the brain? Iron accumulates in specific regions of the brain (primarily the globus pallidus and the substantia nigra) in an age-dependent manner, and iron-induced oxidative damage is implicated in age-dependent neuronal loss (Bartzokis et al., 2004; Zecca et al., 2004a,b; Xu et al., 2010). Moreover, loss of mitochondrial aconitase activity, an enzyme of the citric acid cycle that requires a [4Fe–4S] cluster for function and stability (Bulteau et al., 2003), is a marker of aging (Longo et al., 1999; Bota et al., 2002; Bulteau et al., 2006). Thus, it is possible to once again envision how the need to handle redox-active iron could subject the ISC assembly machinery to progressive accumulation of oxidative damage. This could lead to an age-dependent decline in ISC synthesis and loss of critical ISC enzyme activities, with progressive cellular and mitochondrial iron dysregulation, similar to what we have discussed above for FRDA and PD. **Figure 5** shows a possible unifying model whereby a continuum of clinical phenotypes are associated with ISC synthesis defects of varying degree. We hypothesize that genetically-determined or acquired (environmental or age-dependent) defects in ISC synthesis contribute more prominently to human disease than it is currently appreciated. Indeed, age-dependent accumulation of iron and iron-catalyzed oxidative damage have been implicated not only in FRDA and PD but also Alzheimer’s disease (Zecca et al., 2004b), atherosclerosis (Zacharski et al., 2000), and heart disease (Wood, 2004). As the aging population continues to expand, it is likely that the prevalence of conditions associated with age-dependent iron accumulation will also increase.

## IRON CHELATION FOR TREATMENTS OF FRDA AND PD

As we have seen, eukaryotic cells require mitochondrial ISC synthesis not only to ensure the biogenesis and function of a large number of ISC-dependent enzymes but also to maintain a balance between iron uptake and iron utilization (**Figure 3**). While there are no approaches as yet available to correct defects in ISC synthesis directly, targeting chelatable iron represents a reasonable approach to limit the consequences of these defects, primarily mitochondrial iron overload and iron-catalyzed Fenton chemistry. Iron chelation is widely used to reduce iron deposition in the organs of patients affected by disorders characterized by global iron overload (e.g., hemochromatosis; Barton, 2007; Flaten et al., 2012). However, in the case of FRDA or PD, the regional nature of the iron accumulation and the abnormal iron distribution within the affected cell types have suggested the need for agents able to appropriately “relocate” iron while at the same time limiting its participation in radical-generating reactions (Boddaert et al., 2007). Deferiprone, a chelator used for treating iron overload, has been shown to possess this iron “relocating” ability by scavenging labile iron from mitochondria and delivering it to cytoplasmic and extracellular apotransferrin. In cells derived from FRDA patients, deferiprone decreased the levels of mitochondrial labile iron and also limited oxidative damage (Kakhlon et al. 2008, 2010), presumably by limiting iron-catalyzed Fenton chemistry (Kontoghiorghes, 2009). A six-month open-label single-arm study with deferiprone, administered together with the antioxidant idebenone, was initially conducted in nine adolescents affected by FRDA. By use of magnetic resonance imaging, selective iron removal was observed in the nucleus dentatus of the cerebellum in all patients, while improved neuromotor function was observed in the youngest patients (Boddaert et al., 2007). An 11 month open-labeled study in 20 FRDA patients confirmed that deferiprone and idebenone combined could improve iron deposits in the dentate nucleus with a stabilizing effect on certain neurological parameters (in addition to significantly improved heart hypertrophy parameters; Velasco-Sanchez et al., 2011). However, a subsequent double-blind, randomized placebo-controlled phase 2 trial showed a worsening of ataxia with doses of deferiprone ≥40 mg/Kg/day. In the same study, there were no significant changes in ataxia with lower doses of deferiprone, though improvements in posture, gait, and kinetic function were noted in some patients, and a significant decrease in left ventricular mass in most patients [reviewed in (Wilson, 2012; Pandolfo and Hausmann, 2013)]. These dose-dependent effects may be explained by a report that concentrations of deferiprone ≥50 μM in cultured cells induced iron depletion rather than redistribution, with deleterious effects on ISC enzyme activities and even on frataxin levels (Pandolfo and Hausmann, 2013). A moderate iron chelation regimen that did not alter systemic iron levels was recently tested in a pilot clinical study of deferiprone in early-stage PD patients; this regimen was shown to improve iron deposits in the substantia nigra as well as motor parameters of disease progression (Devos et al., 2013). These studies underscore the importance of identifying iron chelation modalities that can correct iron distribution within affected neuronal cells without inducing deleterious global changes in iron metabolism, including changes in the expression of frataxin and other mitochondrial proteins involved in ISC synthesis. Better understanding of disease natural history and ability to intervene in the pre-symptomatic phase may also be critical to achieve effective treatments.

## CONCLUSION AND FUTURE DIRECTIONS

The best characterized human condition linked to abnormal mitochondrial ISC synthesis is FRDA. Tissue-specific defects in mitochondrial ISC synthesis have more recently been identified in patients with isolated myopathy (Mochel et al., 2008; Olsson et al., 2008) or sideroblastic anemia (Camaschella et al., 2007), which are linked to mutations in the scaffold ISCU and the enzyme glutaredoxin 5, respectively. The combination of genetic and clinical heterogeneity (as illustrated by these three disorders) can make inherited defects in ISC synthesis difficult to recognize, and it is likely that their combined prevalence is underestimated. It is also possible that tissue-specific decline in ISC synthesis is implicated in the iron accumulation that occurs not only in PD and the aging brain (Zecca et al., 2004b) but also the vasculature (Zacharski et al., 2000) and the heart (Wood, 2004) during aging. Another possible link to aging is suggested by a recent report that impaired ISC biogenesis leads to nuclear genome instability (Veatch et al., 2009). These interesting links remain largely unexplored due to the lack of suitable technology. The activities of natural (e.g., mitochondrial aconitase or succinate dehydrogenase; Foury, 1999; Mochel et al., 2008) or artificial (Hoff et al., 2009a,b) ISC-containing enzymes are currently used as measures of ISC synthesis. One limitation is that these activities lay downstream of the initial and rate-limiting step in ISC synthesis (i.e., the assembly of [2Fe–2S] clusters on ISC scaffolds). In addition, they can be influenced by factors (e.g., oxidative stress) independent of the actual rate of ISC synthesis. In essence, technology to interrogate ISC synthesis directly *in vivo*, for translational and clinical research studies, is currently lacking. Development of such technology will open the possibility to (i) assess the extent to which this pathway contributes to neurodegenerative disease and age-related disorders, and (ii) develop strategies to correct primary or secondary functional deficits. Given the role played by ISC synthesis in the maintenance of many vital enzymes and iron homeostasis, as well as the prevention of iron-catalyzed oxidative stress, we predict that this technology will have important biomedical applications.

## Conflict of Interest Statement

Mayo Clinic has a financial interest associated with technology used in the author’s research, which has been licensed to a commercial entity. Mayo Clinic, but not the author, has received royalties of less than the federal threshold for significant financial interest.
